# Validation of sputum Gram stain for treatment of community-acquired pneumonia and healthcare-associated pneumonia: a prospective observational study

**DOI:** 10.1186/1471-2334-14-534

**Published:** 2014-10-18

**Authors:** Hajime Fukuyama, Shin Yamashiro, Kiyoshi Kinjo, Hitoshi Tamaki, Tomoo Kishaba

**Affiliations:** Department of Respiratory Medicine, Okinawa Chubu Hospital, 281 Miyazato, Uruma, Okinawa, Japan; Department of General Internal Medicine, Okinawa Chubu Hospital, 281 Miyazato, Uruma, Okinawa, Japan

**Keywords:** Pneumonia, Sputum, Gram stain, Community-acquired pneumonia, Healthcare-associated pneumonia

## Abstract

**Background:**

The usefulness of sputum Gram stain in patients with community-acquired pneumonia (CAP) is controversial. There has been no study to evaluate the diagnostic value of this method in patients with healthcare-associated pneumonia (HCAP). The purpose of this study was to evaluate the usefulness of sputum Gram stain in etiological diagnosis and pathogen-targeted antibiotic treatment of CAP and HCAP.

**Methods:**

We conducted a prospective observational study on hospitalized patients with pneumonia admitted to our hospital from August 2010 to July 2012. Before administering antibiotics on admission, Gram stain was performed and examined by trained physicians immediately after sputum samples were obtained. We analyzed the quality of sputum samples and the diagnostic performance of Gram stain. We also compared pathogen-targeted antibiotic treatment guided by sputum Gram stain with empirical treatment.

**Results:**

Of 670 patients with pneumonia, 328 were CAP and 342 were HCAP. Sputum samples were obtained from 591 patients, of these 478 samples were good quality. The sensitivity and specificity of sputum Gram stain were 62.5% and 91.5% for *Streptococcus pneumoniae*, 60.9% and 95.1% for *Haemophilus influenzae*, 68.2% and 96.1% for *Moraxella catarrhalis*, 39.5% and 98.2% for *Klebsiella pneumoniae*, 22.2% and 99.8% for *Pseudomonas aeruginosa*, 9.1% and 100% for *Staphylococcus aureus*. The diagnostic yield decreased in patients who had received antibiotics or patients with suspected aspiration pneumonia. Pathogen-targeted treatment provided similar efficacy with a decrease in adverse events compared to empirical treatment.

**Conclusions:**

Sputum Gram stain is highly specific for the etiologic diagnosis and useful in guiding pathogen-targeted antibiotic treatment of CAP and HCAP.

**Electronic supplementary material:**

The online version of this article (doi:10.1186/1471-2334-14-534) contains supplementary material, which is available to authorized users.

## Background

The sputum Grain stain is a simple and inexpensive method for the rapid diagnosis of microbial etiologies of pneumonia. However, the usefulness of sputum Gram stain in the initial approach to patients with community-acquired pneumonia (CAP) is still controversial. Some studies have had doubt on the usefulness of sputum Gram stain in terms of difficulty to obtain good quality samples, sensitivity, reliability, and overall impact on treatment decisions [1–3]. Guidelines do not recommend routine sputum Gram stain on patients with CAP [4–7]. The Japanese Respiratory Society (JRS) guidelines recommends pathogen-specific treatment using rapid diagnostic methods such as sputum Gram stain if possible [8]. However, this treatment strategy has not been validated.

Healthcare-associated pneumonia (HCAP) is a relatively new category of pneumonia proposed by the 2005 American Thoracic Society (ATS)/Infectious Diseases Society of America (IDSA) guidelines [9]. HCAP is distinct from CAP because which has risk factors for multidrug-resistant (MDR) pathogens that often carry a poor prognosis. Thus far no study has evaluated the usefulness of sputum Gram stain in patients with HCAP.

We therefore conducted a prospective study to assess the usefulness of sputum Gram stain on hospitalized patients with CAP and HCAP. Primary objective of our study was the diagnostic performance of sputum Gram stain. The secondary objective was to assess the effectiveness of the initial antibiotic treatment guided by sputum Gram stain.

## Methods

We conducted a prospective observational study of consecutive patients with pneumonia who were hospitalized at Okinawa Chubu Hospital (a 550-bed acute care hospital in Okinawa, Japan) from August 2010 to July 2012. Pneumonia was defined as a new infiltrate on chest X-ray together with signs and symptoms of a lower respiratory tract infection: fever, cough, sputum, dyspnea, chest pain. We excluded patients if they were considered at follow up to have other diseases that distinguished them from pneumonia. Sputum Gram stain was performed and interpreted by trained physicians in the emergency room on admission. We analyzed the diagnostic performance of the sputum Gram stain. We also compared pathogen-targeted antibiotic treatment guided by sputum Gram stain with empirical treatment. This study was approved by ethics committee of Okinawa Chubu Hospital. Sputum samples were collected as part of standard patient care and as this was an observational study, written informed consent was deemed unnecessary.

### Data collection

We collected data on age, sex, onset location, social history, co-morbid conditions, medications, results of laboratory testing, and chest radiographs. We calculated the Pneumonia Severity Index (PSI) of the Patient Outcomes Research Team (PORT score) at admission [10]. We recorded any initial treatment failure, any adverse event of the initial antibiotics, need for intensive care unit (ICU) admission, durations of intravenous antibiotic treatment, length of hospital stay, and in-hospital mortality.

### Sputum evaluation

**Figure 1:**
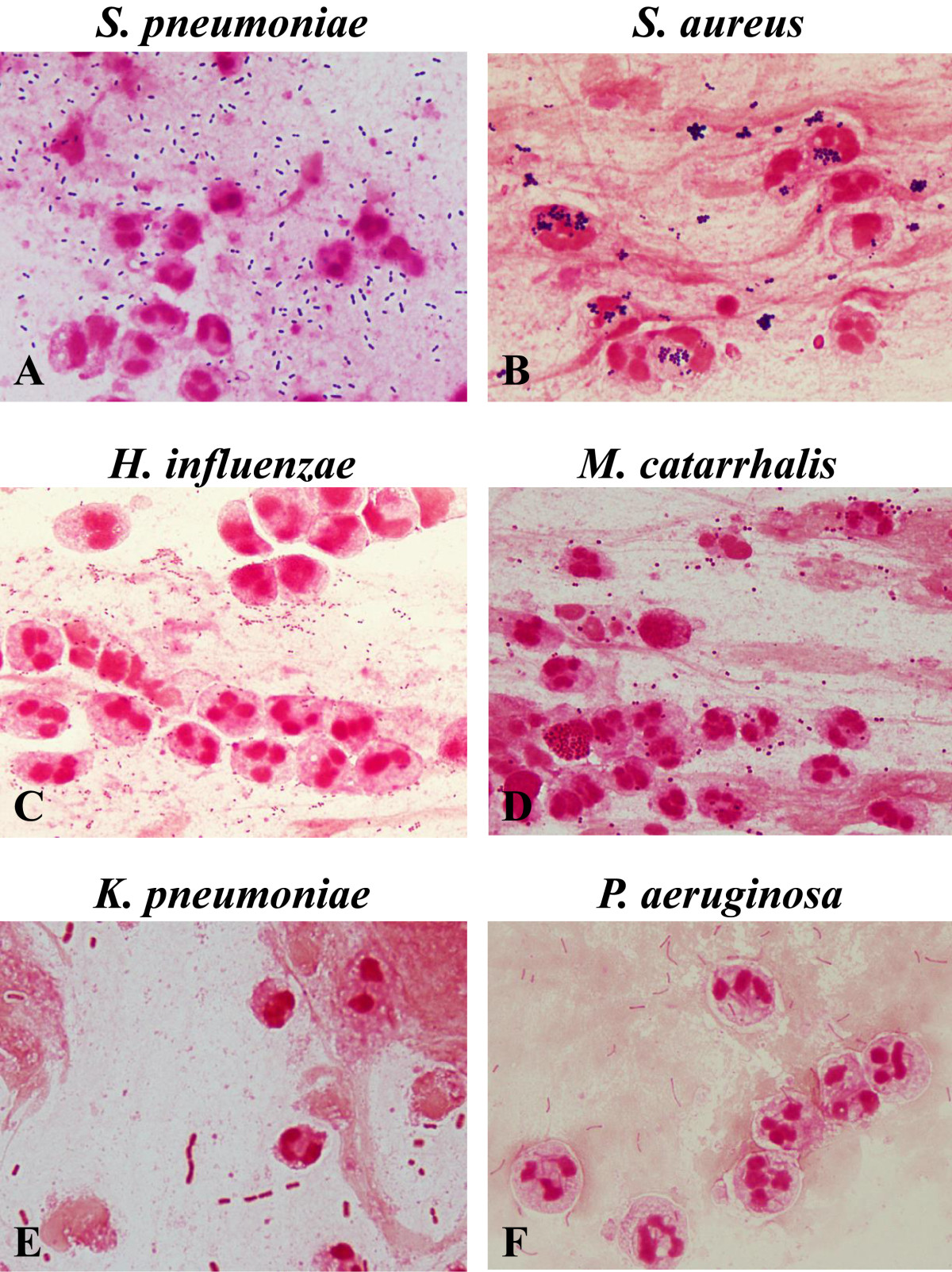
**Bacterial morphotypes in sputum Gram stain (×100, oil immersion field).** Gram positive diplococci (lancet-shaped or football-shaped) are suggestive of *Streptococcus pneumoniae* **(A)**. Cluster of Gram positive cocci are suggestive of *Staphylococcus aureus* **(B)**. Tiny Gram negative coccobacilli are suggestive of *Haemophilus influenzae* **(C)**. Gram negative diplococci (kidney bean-shaped) are suggestive of *Moraxella catarrhalis* **(D)**. Plump Gram negative rods are suggestive of *Klebsiella pneumoniae* **(E)**. Thin gram negative rods are suggestive of *Pseudomonas aeruginosa* **(F)**.

Expectorated sputum samples were collected before administering antibiotics. Nasotracheal suctioned sputum samples were collected by the attending nurse from the patients who could not expectorate due to altered mental status. The Gram stain was performed and interpreted by trained resident physicians (Post-graduate year 1 or 2) as soon as possible after the sputum samples were obtained. Sputum samples were considered of good quality if they had <10 squamous epithelial cells (SECs) per low-power field (LPF) and >10 polymorphonuclear leukocytes (PMNs) per oil immersion field (OIF). Other samples were excluded from the evaluation. In good quality samples, >10 microorganisms of same morphotype at OIF were considered as meaningful. The presence of many morphologic microorganisms which a predominant morphotype was not identified was considered as polymicrobial flora. Morphotypes and the presumptive bacteria were following (Figure 1): Gram-positive lancet-shaped diplococci (GPDC) for *Streptococcus pneumoniae*, Gram-negative coccobacilli (GNCB) for *Haemophilus influenzae*, Gram-negative diplococci (GNDC) for *Moraxella catarrhalis*, Gram-negative rods large sized (GNR-large) for *Klebsiella pneumoniae*, Gram-negative rods small sized (GNR-small) for *Pseudomonas aeruginosa*, Gram-positive cocci in clusters (GPC-cluster) for *Staphylococcus aureus*.

### Reference standard

There is no universally accepted gold standard for assessing the diagnostic value of sputum Gram stain. Previous studies used the sputum culture or blood culture. Although sputum culture is most commonly used reference standard, it lacks both sensitivity and specificity [11, 12]. Blood culture is highly specific but poorly sensitive [13, 14]. Some authors reported that investigation of sputum by a combination of Gram stain, culture, and detection pneumococcal antigen was the most useful means of establishing an aetiological diagnosis of CAP [15]. In our study, the combination of multiple diagnostic methodologies was used as reference standard because we consider it reliable. An etiological diagnosis was considered presumptive if any of the following criteria were fulfilled: 1) moderate to heavy growth from semiquantitative sputum culture; 2) positive culture in bronchoalveolar lavage or pleural fluid; 3) positive blood culture if no other source was identified; 4) positive urinary antigen test for *S. pneumoniae*. Two sets of blood cultures were performed before administering antibiotics in all patients. Bronchoalveolar lavage samples or pleural fluid samples were collected when clinically indicated.

### Definitions

Patients with pneumonia were classified into CAP and HCAP. HCAP included any patients who was 1) hospitalized in an acute care hospital for two or more days within the past 90 days, 2) resided in a nursing home or long-term care facility, 3) received recent intravenous antibiotic therapy, chemotherapy, or wound care within the past 30 days, 4) attended a hospital or hemodialysis clinic [9]. Patients were classified into CAP if they did not meet the criteria for HCAP.

Patients were defined as being immunosuppressed if they had more than one of the following risk factors: 1) daily administration of systemic corticosteroids (at least 10 mg per day of prednisone or an equivalent drug); 2) administration of an immunosuppressive drug; 3) received cancer chemotherapy within the past 30 days; 4) recipient of transplantation (bone marrow or solid organ); 5) underlying congenital or acquired immune deficiency disorder.

The diagnosis of aspiration pneumonia was made based on the JRS guidelines for the management of hospital-acquired pneumonia [16]: overt aspiration (apparent aspiration), a condition in which aspiration was strongly suspected, or the existence of abnormal swallowing function or dysphagia. In addition, we carried out water swallowing tests or videoendoscopy for the purpose of swallowing function evaluation in suspected cases.

The initial antibiotic treatment was considered as being pathogen-targeted if ampicillin was prescribed to a patient with GPDC (*S. pneumoniae*) on the Gram stain, a third-generation cephalosporin was prescribed to a patient with GNCB (*H. influenzae*), ampicillin-sulbactam was prescribed to a patient with GNDC (*M. catarrhalis*), a second or third-generation cephalosporin was prescribed to a patient with GNR-large (*K. pneumoniae*), an antipseudomonal agent was prescribed to a patient with GNR-small (*P. aeruginosa*), and vancomycin was prescribed to a patient with GPC-cluster (*S. aureus*).

### Statistical analysis

For the comparisons between groups, we used the χ2 or Fisher exact test for categorical variables and Mann–Whitney U test for continuous variables. Statistical significance was defined as p < 0.05. The performance of sputum Gram stain was evaluated compared with reference standard. Diagnostic parameters such as sensitivity, specificity, and positive predictive value (PPV) and negative predictive value (NPV) were calculated. All data were analyzed and processed on Stata 11® (StataCorp, College Station, TX, USA).

## Results

### Characteristics of patients

The characteristics of patients with CAP and HCAP are listed in Table 1. A total of 670 patients with pneumonia were enrolled in this study. Of these, 328 had CAP and 342 had HCAP. The median age was 77 years (interquartile range (IQR) 65–85 years). Six hundred thirty five patients (94.8%) had at least one underlying disease; chronic lung disease 301, chronic heart disease 152, chronic liver disease 21, chronic kidney disease 55, cerebrovascular disease 180, neuromuscular disease 64, cancer 58, collagen vascular disease 15, dementia 97, diabetes mellitus 128, hypertension 305, and hyperlipidemia 92. An etiological diagnosis was established in 417 (62.2%) of 670 patients. *S. pneumoniae* was the most frequent causative pathogen in both CAP and HCAP.

**Table 1:** **Characteristics of patients with CAP and HCAP**

	All patients	CAP	HCAP
	n = 670	n = 328	n = 342
Patient background			
Age, median (IQR)	77 (65–80)	75 (59–83)	80 (72–87)
Male, n (%)	430 (64.2)	212 (64.6)	218 (63.7)
Comorbid conditions, n (%)	635 (94.8)	296 (90.2)	339 (99.1)
Previous antibiotics treatment, n (%)	97 (14.5)	34 (10.4)	63 (18.4)
Immunosuppressed, n (%)	40 (6.0)	19 (5.8)	21 (6.1)
Suspected aspiration, n (%)	246 (36.7)	50 (15.2)	196 (57.3)
Severity scores			
PSI score, median (IQR)	110 (89–140)	96 (73–120)	125 (104–153)
PSI class, median (IQR)	4 (3–5)	4 (3–4)	4 (4–5)
Clinical outcomes			
Initial treatment failure, n (%)	78 (11.6)	28 (8.5)	50 (14.6)
Antibiotics adverse effect, n (%)	40 (6.0)	19 (5.8)	21 (6.1)
ICU admission, n (%)	81 (12.1)	41 (12.5)	40 (11.7)
Length of antibiotic treatment, median (IQR)	8 (6–11)	7 (6–10)	9 (7–13)
Length of hospital stay, median (IQR)	11 (8–19)	9 (7–16)	12 (9–22)
In-hospital mortality, n (%)	59 (8.8)	19 (5.8)	30 (8.8)
Pathogen identified, n (%)	417 (62.2)	206 (62.8)	211 (61.7)
*Streptococcus pneumoniae,* n (%)	139 (20.7)	76 (23.2)	63 (18.4)
*Haemophilus influenzae,* n (%)	122 (18.2)	61 (18.6)	61 (17.8)
*Moraxella catarrhalis,* n (%)	41 (6.1)	20 (6.1)	21 (6.1)
*Klebsiella pneumoniae,* n (%)	43 (6.4)	11 (3.4)	32 (9.4)
*Pseudomonas aeruginosa,* n (%)	29 (4.3)	12 (3.7)	17 (5.0)
*Staphylococcus aureus,* n (%)	11 (1.6)	2 (0.6)	9 (2.6)
*Mycoplasma pneumoniae,* n (%)	10 (1.5)	9 (2.7)	1 (0.3)
*Chlamydophila pneumoniae,* n (%)	8 (1.2)	7 (1.0)	1 (0.3)
*Legionella pneumophila,* n (%)	2 (0.3)	0	2 (0.6)

### Sputum samples evaluation

**Figure 2:**
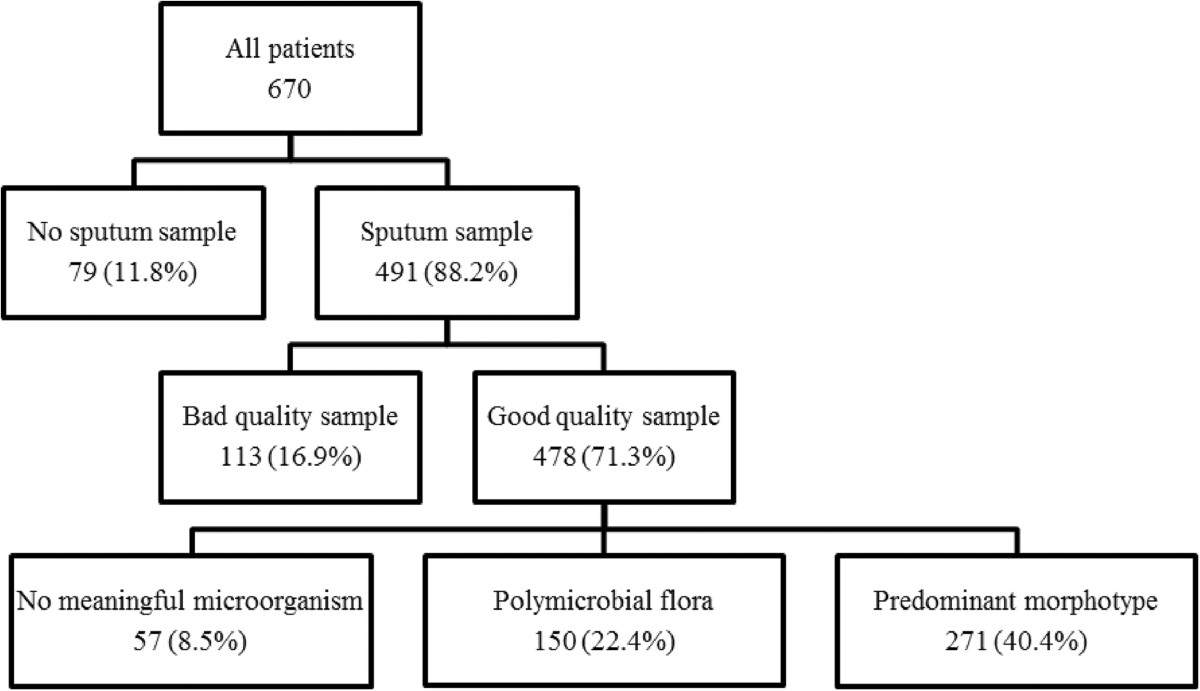
**Results of sputum Gram stain in patients with CAP and HCAP.**

As shown in Figure 2 and Table 2, sputum samples were obtained from 591 patients. Of the samples, 478 were considered of good quality. Two hundred seventy one showed a predominant morphotype, 150 showed polymicrobial flora, and 57 showed no meaningful microorganisms. In patients who had received antibiotics before admission, the sputum samples showed no meaningful microorganism more frequently (24.7 vs 5.8%, p < 0.0001) and lower diagnostic yield (13.4 vs 30.7%, p = 0.0005) than those who had not. In patients with suspected aspiration pneumonia, sputum samples showed polymicrobial flora more frequently (33.7 vs 15.8%, p < 0.0001) with a lower diagnostic yield (20.7 vs 32.5%, p = 0.0011) than those without aspiration.

**Table 2:** **Sputum samples evaluation according to patient backgrounds**

	All patients	Previous antibiotics	Immunosuppressed	Suspected aspiration
	n = 670	n = 97	n = 40	n = 246
No sputum sample, n (%)	79 (11.8)	11 (11.3)	7 (17.5)	5 (2.0)
Poor quality sample, n (%)	113 (16.9)	20 (20.6)	9 (22.5)	54 (22.0)
Good quality sample, n (%)	478 (71.3)	66 (68.0)	24 (60.0)	187 (76.0)
No meaningful microorganism, n (%)	57 (8.5)	24 (24.7)	2 (5.0)	25 (10.2)
Polymicrobial flora, n (%)	150 (22.4)	16 (16.5)	4 (10.0)	83 (33.7)
Predominant morphotype, n (%)	271 (40.4)	26 (26.8)	18 (45.0)	79 (32.1)
Positive predict, n (%)	189 (28.2)	13 (13.4)	9 (22.5)	51 (20.7)

### Predictive accuracy for the etiologic diagnosis

Table 3 shows the predictive performance of sputum Gram stain according to predominant morphotype. The sensitivity and specificity of sputum Gram stain were 62.5% and 91.5% for *S. pneumoniae*, 60.9% and 95.1% for *H. influenzae*, 68.2% and 96.1% for *M. catarrhalis*, 39.5% and 98.2% for *K. pneumoniae*, 22.2% and 99.8% for *P. aeruginosa*, 9.1% and 100% for *S. aureus*.

**Table 3:** **Predictive accuracy of sputum Gram stain for the etiologic diagnosis**

	Sensitivity	Specificity	PPV	NPV	LR (+)	LR (-)
GPDC (*Streptococcus pneumoniae*)	62.5 (70/112)	91.5 (335/366)	69.3 (70/101)	88.9 (335/377)	7.38	0.41
GNCB (*Haemophilus influenzae*)	60.9 (67/110)	95.1 (350/368)	78.8 (67/85)	89.1 (350/393)	12.5	0.41
GNDC (*Moraxella catarrhalis*)	68.2 (30/44)	96.1 (417/434)	63.8 (30/47)	96.8 (417/431)	17.4	0.33
GNR-large (*Klebsiella pneumoniae*)	39.5 (15/38)	98.2 (432/436)	65.2 (15/23)	95.0 (432/455)	21.7	0.62
GNR-small (*Pseudomonas aeruginosa*)	22.2 (6/27)	99.8 (450/451)	85.7 (6/7)	95.5 (450/471)	100.2	0.78
GPC-cluster (*Staphylococcus aureus*)	9.1 (1/11)	100 (467/467)	100 (1/1)	97.1 (467/477)	-	0.91

### Diagnostic value in patients with HCAP

Table 4 shows the diagnostic performance of sputum Gram stain in patients with CAP and HCAP. The diagnostic yield in HCAP patients was lower than that in CAP patients (24.0 vs 32.6%, p = 0.0129). The specificity for the etiologic diagnosis was high in both CAP and HCAP. The sensitivity for the etiologic diagnosis in HCAP was lower than that in CAP.

**Table 4:** **Diagnostic performance of sputum Gram stain in patients with CAP and HCAP**

	CAP	HCAP	p value
	n = 328	n = 342	
No sputum sample, n (%)	55 (16.8)	24 (7.0)	<0.001
Poor quality sample, n (%)	43 (13.1)	70 (20.5)	0.011
Good quality sample, n (%)	230 (70.1)	248 (72.5)	0.49
No meaningful microorganism, n (%)	30 (9.1)	27 (7.9)	0.56
Polymicrobial flora, n (%)	56 (17.1)	94 (27.5)	0.001
Predominant morphotype, n (%)	144 (43.9)	127 (37.1)	0.0744
Positive predict, n (%)	107 (32.6)	82 (24.0)	0.013
Sensitivity (%)			
*Streptococcus pneumoniae*	63.1	61.7	
*Haemophilus influenzae*	76.8	44.4	
*Moraxella catarrhalis*	85.0	54.2	
*Klebsiella pneumoniae*	50.0	37.5	
*Pseudomonas aeruginosa*	20.0	23.5	
*Staphylococcus aureus*	50.0	0	
Specificity (%)			
*Streptococcus pneumoniae*	89.7	93.0	
*Haemophilus influenzae*	94.8	95.3	
*Moraxella catarrhalis*	97.6	92.4	
*Klebsiella pneumoniae*	98.7	99.5	
*Pseudomonas aeruginosa*	100	99.6	
*Staphylococcus aureus*	100		

### Pathogen-targeted treatment and empirical treatment

Comparison between pathogen-targeted treatment and empirical treatment is shown in Table 5. Among the 271 patients with a predominant morphotype, 174 patients received pathogen-targeted initial antibiotic treatment. Severity scores were higher in patients who received empirical treatment than in patients who received pathogen-targeted treatment. There was no significant difference regarding the frequency of initial antibiotic treatment failure and in-hospital mortality between the two groups. The frequency of adverse events was significantly lower in patients who received pathogen-targeted treatment (2.9 vs 7.0%, p = 0.0492). The most frequent adverse event was skin rash (n = 18), followed by pseudomembranous enterocolitis (n = 8), elevated liver enzymes (n = 8), drug fever (n = 3), thrombocytopenia (n = 3). Length of hospital stay and length of intravenous antibiotic therapy were significantly longer in patients who received empirical treatment.

**Table 5:** **Comparison between pathogen-targeted treatment and empirical treatment**

	Pathogen-targeted treatment	Empirical treatment	p value
	n = 172	n = 498	
Patient background			
Age, median (IQR), *y*	80 (72–88)	76 (63–85)	0.064
Male, n (%)	90 (52.3)	340 (68.3)	<0.001
Comorbid conditions, n (%)	163 (94.8)	472 (94.8)	0.157
CAP, n (%)	100 (58.1)	228 (45.8)	0.005
Previous antibiotics treatment, n (%)	14 (8.1)	83 (16.7)	0.006
Immunosuppressed, n (%)	8 (4.7)	32 (6.4)	0.40
Suspected aspiration, n (%)	43 (25.0)	203 (40.8)	<0.001
Severity scores			
PSI score, median (IQR)	108 (89–128)	112 (90–142)	0.012
PSI class, median (IQR)	4 (3–4)	4 (4–5)	0.002
Clinical outcomes			
Initial treatment failure, n (%)	13 (7.6)	61 (12.2)	0.091
Antibiotics adverse events, n (%)	5 (2.9)	35 (7.0)	0.049
ICU admission, n (%)	12 (7.0)	69 (13.9)	0.017
Length of intravenous treatment, median(IQR)	8 (6–9)	9 (7–13)	<0.001
Length of hospital stay, median (IQR)	9 (7–13)	11 (8–21)	<0.001
In-hospital mortality, n (%)	14 (8.1)	45 (9.0)	0.72

## Discussion

The usefulness of sputum Gram stain in patients with CAP is controversial. While some authors have insisted on the usefulness of sputum Gram stain [17–19], others have argued for a limited value of this method [1–3]. The diagnostic performance of sputum Gram stain in CAP varies in different studies. The meta-analysis which evaluated the sputum Gram stain in community-acquired pneumococcal pneumonia showed that the sensitivity ranged 15-100% and specificity from 11-100% [20]. This might be due to variations in study methodology. We should take into account that the diagnostic yield of sputum Gram stain depends on the reference standards, the definitions of the positive Gram stain, and the population of patients.

Some authors have pointed out that the limited value of sputum Gram stain is due to the difficulty to obtain a good quality sample [21, 22]. In our study, a good quality sputum samples was obtained in 478 (71.3%) of 670 patients, which is a higher yield than reported in previous studies [21, 22]. One reason for this is that we attempted to collect not only expectorated sputum samples but also nasotracheal suctioned sputum samples. We consider it a reflection of our clinical practice that nasotracheal suctioning from patients who could not cough up sputum due to altered mental status was usually performed. Another reason is the rapid collection and processing of the sputum samples. Our hospital has a house staff laboratory in the emergency room, and physicians can perform Gram stain immediately after samples are obtained. One study reported that good quality samples had been obtained in only 20% of all patients, but the delay in collection and laboratory processing of the samples was considerable [3]. The IDSA/ATS guidelines recommends that Gram stain should be performed only if quality performance measures for collection, transport, and processing of samples can be met [4].

Previous studies reported that receiving antibiotics before sputum sample collection adversely affect the performance of Gram stain [3, 18, 22]. Our results also demonstrated that previous antibiotic treatment decreased the diagnostic yield. Sputum samples without identification of a meaningful microorganism were more frequently obtained from patients who had received antibiotics. In addition, we found that patients with suspected aspiration pneumonia often showed polymicrobial flora on sputum Gram stain more frequently and lower diagnostic yield. Samples collected from patients with aspiration pneumonia are often contaminated by the flora of the oropharynx or upper airway.

The diagnostic value of sputum Gram stain for *S. pneumoniae* or *H. influenzae* was reported in many previous studies [17–22]. However, other causative bacteria for pneumonia can be estimated on sputum Gram stain. We also investigated for *S. aureus*, *M. catarrhalis*, *P. aeruginosa*, and *K. pneumoniae* in this study. The sputum Gram stain was highly specific (>90%) for the diagnosis of the all bacteria. These results suggest that a positive sputum Gram stain can lead to appropriate initial antibiotic selection. On the contrary, the sensitivity varied in different bacteria. The sensitivity for *S. aureus*, *P. aeruginosa*, *K. pneumoniae* was low (9.1 to 39.5%). Failure to detect these bacteria on sputum Gram stain does not mean the absence.

To our knowledge, this is the first study to evaluate the usefulness of sputum Gram stain in patients with HCAP. Although the 2005 IDSA/ATS guidelines recommended that all HCAP patients need broad-spectrum antibiotic treatment [9], recent reports showed that this approach is not appropriate because not all HCAP patients had MDR pathogens [23, 24]. The therapeutic strategy for the appropriate initial antibiotic treatment without overuse of broad-spectrum antibiotics in HCAP patients is needed. We found the diagnostic yield of sputum Gram stain in HCAP patients was lower than that in CAP patients. The reason for this is that HCAP included more patients with suspected aspiration pneumonia. However, in good quality samples, the Gram stain was highly specific (>90%) for the etiologic diagnosis in HCAP patients. These results indicate that sputum Gram stain is useful in guiding pathogen-targeted treatment for HCAP patients.

Pathogen-targeted treatment guided by sputum Gram stain provided similar efficacy with less frequent adverse events than empirical treatment in this study. These results were similar to those of a randomized control trial in which a pathogen directed approach was compared with empirical treatment in patients with CAP [25]. Adverse events of antibiotics often lead to poor outcomes such as increased in-hospital mortality and longer length of hospital stay. A recent study reported that empirical treatment in concordance with the ATS/IDSA guidelines was associated with increased mortality in hospital-acquired pneumonia (HAP) and HCAP [26]. A potential explanation could be antibiotic-specific adverse effect. Additionally, the most important advantage of pathogen-targeted treatment is a reduction in antimicrobial resistance [27]. While there is no sufficient evidence of a causative association between pathogen-targeted treatment and antimicrobial resistance, there is little doubt that appropriate antibiotic stewardship can reduce the resistance. Physicians should make an effort to identify the cause of pneumonia and avoid inappropriate overuse of broad-spectrum antibiotics.

One of the notable features in our study method is that the Gram stain was performed and interpreted by physicians rather than laboratory technicians. Gram staining by physicians is routine clinical practice in our hospital. The resident physicians have been trained the microbial examination as a part of our postgraduate medical education. A benefit of Gram staining by physicians is that they can check the quality of sputum samples and the bacteria with their own eyes. The information is useful for estimating causative bacteria in the initial approach, and often leads to pathogen-oriented treatment. We found 174 of 271 (64.2%) patients with predominant morphotype on Gram stain were treated with pathogen-targeted treatment.

An additional benefit of sputum Gram stain is that it can validate the subsequent sputum culture results [4]. The growth of an organism from a sputum sample does not always indicate the presence of infection. Sputum samples can become contaminated with saliva or upper respiratory tract flora. Results of sputum culture can yield false positive findings related to colonization or contamination, and thus should be utilized with the results of the sputum Gram stain when establishing the definitive etiologic diagnosis of pneumonia.

There are several limitations to our study that should be acknowledged. First, this is a single center study. Our availability of Gram stain may not be applied to other hospital settings. The performance of sputum Gram stain is directory related to quality in the processing of samples [3], and experience of the interpreters [28]. In many hospitals, microbiological examination is outsourced and few physicians perform Gram stain [29, 30]. Second, atypical pathogens could not be fully evaluated. Third, this is not a randomized control study to compare pathogen-targeted treatment with empirical treatment. Further study which evaluates the clinical efficacy of pathogen-targeted treatment guided by sputum Gram stain is needed.

## Conclusion

In conclusion, sputum Gram stain is highly specific for the etiologic diagnosis of CAP and HCAP and useful in guiding pathogen-targeted antibiotic treatment.

## Authors’ original submitted files for images

Below are the links to the authors’ original submitted files for images. Authors’ original file for figure 1 Authors’ original file for figure 2
