# Supplementary figures and images for: Validation of sputum Gram stain for treatment of community-acquired pneumonia and healthcare-associated pneumonia: a prospective observational study
Source: BMC Infect Dis. 2014 Oct 18;14:534. doi: 10.1186/1471-2334-14-534 (PMC4287475; doi:10.1186/1471-2334-14-534)

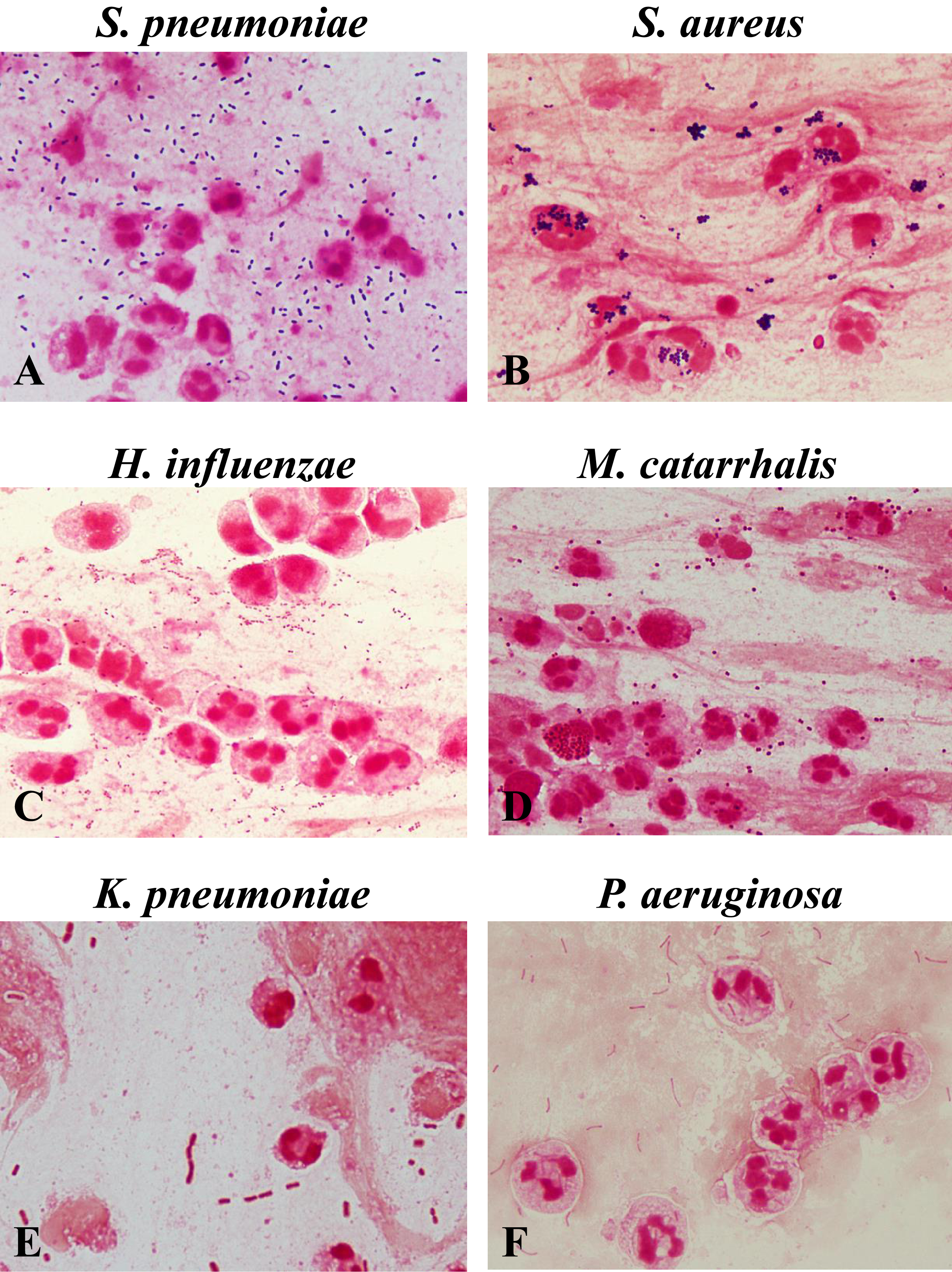

Supplement: Supplementary file 1 — Authors’ original file for figure 1 [file 12879_2014_4001_MOESM1_ESM.tif]

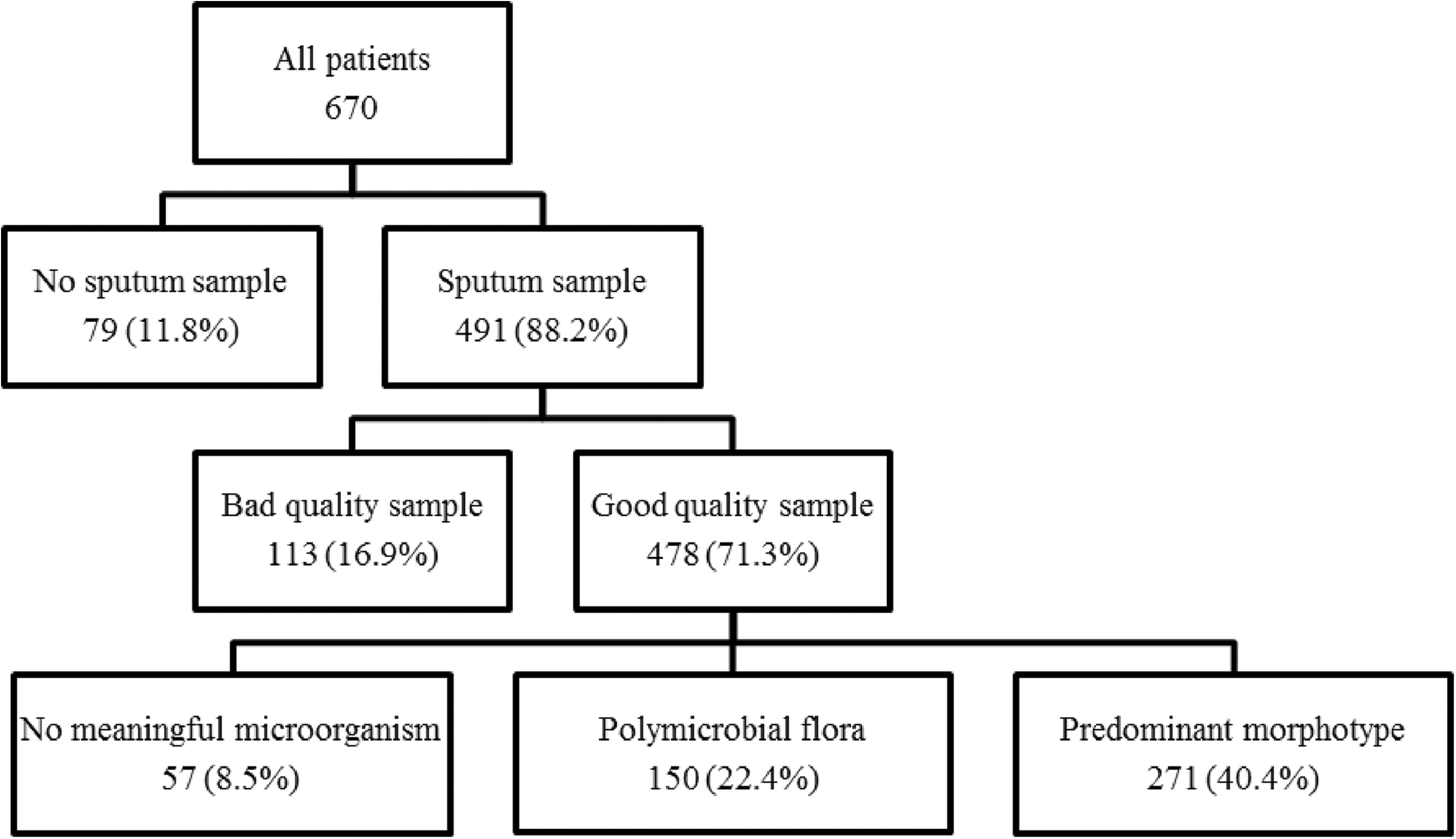

Supplement: Supplementary file 2 — Authors’ original file for figure 2 [file 12879_2014_4001_MOESM2_ESM.tif]
